# Supplementary material for: The effect of the COVID-19 pandemic on the gender gap in research productivity within academia
Source: eLife. 2023 Jul 6;12:e85427. doi: 10.7554/eLife.85427 (PMC10365834; doi:10.7554/eLife.85427)
Supplement: Supplementary file 2. [file elife-85427-supp2.docx]

**Table S1. Naive Boolean search string used in initial literature search.**

Wildcards (*) were used to return results containing different word endings. Texts were limited to those since 2020. Search was conducted on 10/07/2021.

| Concept group (PICO) | Terms |
| --- | --- |
| Academia (population) | ( academi* OR author* OR database* OR journal* OR research OR scien* ) |
| Gender (population) | AND    ( female* OR gender OR male* OR men OR women ) |
| Pandemic (intervention) | AND    ( coronavirus OR covid OR pandemic ) |
| Inequality (comparator) | AND    ( bias* OR disparit* OR disproportion* OR fewer OR gap OR "gender difference*" OR imbalance* OR inequalit* OR inequit* OR parity OR "sex difference*" OR skew* OR unequal ) |
| Productivity (outcome) | AND  ( performan* OR publication* OR publish* OR productiv* ) |
| Exclusion of biomedical studies (population) | AND NOT  ( surviv* OR experiment OR laboratory ) |

**Table S2. Top 60 strongest terms in the term co-occurrence matrix and their rank in ascending order.**

Terms in bold were included in the AND NOT operator concept group excluding biomedical studies.

| Term | Strength | Rank ascending |
| --- | --- | --- |
| child | 4257 | 2063 |
| regression | 4335 | 2064 |
| survey | 4367 | 2065 |
| ***acute respiratory*** | 4427 | 2066 |
| embase | 4637 | 2067 |
| databases | 4646 | 2068 |
| death | 4664 | 2069 |
| knowledge | 4764 | 2070 |
| hip | 4918 | 2071 |
| mortality | 5000 | 2072 |
| female | 5065 | 2073 |
| rights reserved | 5257 | 2074 |
| city | 5504 | 2075 |
| information | 5700 | 2076 |
| symptom | 6108 | 2077 |
| sars-cov-2 | 6112 | 2078 |
| sars-cov | 6185 | 2079 |
| coronavirus disease 2019 | 6397 | 2080 |
| systematic review | 6506 | 2081 |
| rest | 6578 | 2082 |
| science | 6659 | 2083 |
| distance | 6679 | 2084 |
| literature | 6816 | 2085 |
| database | 6916 | 2086 |
| medical | 7023 | 2087 |
| ***icu*** | 7170 | 2088 |
| affect | 7297 | 2089 |
| control | 7386 | 2090 |
| population | 7418 | 2091 |
| women | 7422 | 2092 |
| bias | 7665 | 2093 |
| gender | 7693 | 2094 |
| male | 7977 | 2095 |
| coronavirus disease | 8009 | 2096 |
| article | 8135 | 2097 |
| virus disease | 8230 | 2098 |
| ***iga*** | 8235 | 2099 |
| hospital | 8318 | 2100 |
| work | 8319 | 2101 |
| covid-19 pandemic | 8377 | 2102 |
| ***gis*** | 8489 | 2103 |
| severe | 8518 | 2104 |
| time | 8831 | 2105 |
| infection | 8914 | 2106 |
| ***rna*** | 10303 | 2107 |
| outcome | 10461 | 2108 |
| review | 11869 | 2109 |
| coronavirus | 12243 | 2110 |
| stem | 12316 | 2111 |
| ***risk*** | 12776 | 2112 |
| analysis | 13610 | 2113 |
| research | 13737 | 2114 |
| virus | 13835 | 2115 |
| pandemic | 14212 | 2116 |
| author | 14333 | 2117 |
| car | 15893 | 2118 |
| over | 15964 | 2119 |
| health | 16176 | 2120 |
| covid-19 | 21088 | 2121 |
| age | 21194 | 2122 |
| covid | 21330 | 2123 |

**Table S3. Flowchart of questions used for initial screen of title, abstract and key-words**. Any 'No' answer meant that the study was excluded. 'Yes' AND/OR 'Maybe' answers to all of the below meant that the study was included. Reports such as reviews or comments investigating our PICO framework were included. Studies that lacked a formal abstract but had a title or informal abstract suggesting at least 3 of the above questions were answered with a ‘yes’.

| Initial screen question | Additional notes |
| --- | --- |
| 1) Population: Is there suggestion the study investigates academia/ research/academics/journals? | Include studies that do not specifically investigate academia/research/academics/journals, but considers the effect on ‘work’ or ‘work productivity’ at a broader level, with potentially enough power to subdivide effects on different workplaces, such as academia.  Some academic fields such as those within medicine and engineering are dominated by a practical/applied/professional/labour component. Exclude studies with explicit reference to only investigating the professional/practical/labour context of these fields. Include studies if no reference is made to whether the academic field is investigated in a research or professional capacity. |
| 2) Intervention: Is there suggestion the study investigates the effect of the pandemic? | There is no exact start date to the global pandemic. Include all studies mentioning the pandemic as the exact dates defining the duration of the pandemic will be chosen at the discretion of the authors. |
| 3) Comparison: Is there suggestion the study investigates pre-pandemic? | Often titles and abstracts do not have dates and will not refer explicitly to pre-pandemic data being included. Include studies if there is suggestion the ‘effect of the pandemic’ (or similar) is considered, assume a comparison with pre-pandemic data is included even if no explicit reference to pre-pandemic productivity is considered.  There is no exact start date to the global pandemic. Include all studies mentioning the pandemic as the exact dates defining the duration of time before the pandemic will be chosen at the discretion of the authors. |
| 4) Outcome: Is there suggestion the study investigates the effect on gender in productivity/publication/submission/authorship numbers? | Include studies if there is no reference to investigating the effect of gender or sex, but does refer to investigating broader sociodemographic or socioeconomic factors because gender may be investigated in the full text.    Include studies that suggest investigation of any form of research-related activity. |

**Figure S1. PRISMA flow diagram for number of records included at each stage in first and iterated search.**

| **Identification** |  |  | 10/07/2021 search | |  |  |
| --- | --- | --- | --- | --- | --- | --- |
|  |  | Web of Science | Scopus | EBSCO | ProQuest |  |
|  |  | N=199 | N=126 | N=276 | N=99 |  |
|  |  |  | ↓ | |  |  |
|  |  |  | Total | |  |  |
|  |  |  | N=700 | |  |  |
| **Screening** |  |  | ↓ | |  |  |
|  |  |  | De-duplicated | |  |  |
|  |  |  | N=580 | |  |  |
|  |  |  | ↓ | |  |  |
|  |  |  | Title, abstract, keyword screened | |  |  |
|  |  |  | N=81 | |  |  |
| **Include** |  |  | ↓ | |  |  |
|  |  |  | Full text screened | |  |  |
|  |  |  | N=25 | |  |  |
|  |  |  |  | |  |  |
|  |  |  | 28/02/2022 search | |  |  |
| **Identification** |  | Web of Science | Scopus | EBSCO | ProQuest |  |
|  |  | N=413 | N=258 | N=542 | N=433 |  |
|  |  |  | ↓ | |  |  |
|  |  |  | Total | |  |  |
|  |  |  | N=1646 | |  |  |
|  |  |  | ↓ | |  |  |
| **Screening­­­­** |  |  | De-duplicated | |  |  |
|  |  |  | N=1208 | | → | Excluded initial screen |
|  |  |  | ↓ | |  | N=1039 |
|  |  |  | Title, abstract, keyword screened | |  |  |
|  |  |  | N=169 | | → | Excluded full screen |
|  |  |  | ↓ | |  | Missed 1 qualifier N=53 |
| **Included** | Scoped texts |  | Full text screened | |  | Missed 2 qualifiers N=38 |
|  | N=5 | → | N=50 | |  | Missed 3 qualifiers N=27 |
|  |  |  | ↓ | |  | Missed 4 qualifiers N=1 |
|  |  |  | Total texts in Meta-analysis | |  |  |
|  |  |  | N=55 | |  |  |

**Table S4. PRISMA checklist**

| **Section and Topic** | **Item #** | **Checklist item** | **Where item is reported** |
| --- | --- | --- | --- |
| **TITLE** | | |  |
| Title | 1 | Identify the report as a systematic review. | Page 2 |
| **ABSTRACT** | | |  |
| Abstract | 2 | See the PRISMA 2020 for Abstracts checklist. | Page 2 |
| **INTRODUCTION** | | |  |
| Rationale | 3 | Describe the rationale for the review in the context of existing knowledge. | Page 3 |
| Objectives | 4 | Provide an explicit statement of the objective(s) or question(s) the review addresses. | Page 4 |
| **METHODS** | | |  |
| Eligibility criteria | 5 | Specify the inclusion and exclusion criteria for the review and how studies were grouped for the syntheses. | Pages 20,21 |
| Information sources | 6 | Specify all databases, registers, websites, organisations, reference lists and other sources searched or consulted to identify studies. Specify the date when each source was last searched or consulted. | Pages 20,21 |
| Search strategy | 7 | Present the full search strategies for all databases, registers and websites, including any filters and limits used. | Pages 20,21 & Table 1 |
| Selection process | 8 | Specify the methods used to decide whether a study met the inclusion criteria of the review, including how many reviewers screened each record and each report retrieved, whether they worked independently, and if applicable, details of automation tools used in the process. | Pages 21 |
| Data collection process | 9 | Specify the methods used to collect data from reports, including how many reviewers collected data from each report, whether they worked independently, any processes for obtaining or confirming data from study investigators, and if applicable, details of automation tools used in the process. | Page 21 |
| Data items | 10a | List and define all outcomes for which data were sought. Specify whether all results that were compatible with each outcome domain in each study were sought (e.g. for all measures, time points, analyses), and if not, the methods used to decide which results to collect. | Pages 20,21 |
|  | 10b | List and define all other variables for which data were sought (e.g. participant and intervention characteristics, funding sources). Describe any assumptions made about any missing or unclear information. | Pages 20,21 |
| Study risk of bias assessment | 11 | Specify the methods used to assess risk of bias in the included studies, including details of the tool(s) used, how many reviewers assessed each study and whether they worked independently, and if applicable, details of automation tools used in the process. | NA |
| Effect measures | 12 | Specify for each outcome the effect measure(s) (e.g. risk ratio, mean difference) used in the synthesis or presentation of results. | Pages 20,21 |
| Synthesis methods | 13a | Describe the processes used to decide which studies were eligible for each synthesis (e.g. tabulating the study intervention characteristics and comparing against the planned groups for each synthesis (item #5)). | Page 21, Supplementary file 3 |
|  | 13b | Describe any methods required to prepare the data for presentation or synthesis, such as handling of missing summary statistics, or data conversions. | Page 23, Supplementary file 1 |
|  | 13c | Describe any methods used to tabulate or visually display results of individual studies and syntheses. | Pages 24,25 |
|  | 13d | Describe any methods used to synthesize results and provide a rationale for the choice(s). If meta-analysis was performed, describe the model(s), method(s) to identify the presence and extent of statistical heterogeneity, and software package(s) used. | Pages 24,25 |
|  | 13e | Describe any methods used to explore possible causes of heterogeneity among study results (e.g. subgroup analysis, meta-regression). | Page 25 |
|  | 13f | Describe any sensitivity analyses conducted to assess robustness of the synthesized results. | Page 25 |
| Reporting bias assessment | 14 | Describe any methods used to assess risk of bias due to missing results in a synthesis (arising from reporting biases). | Pages 25,26 |
| Certainty assessment | 15 | Describe any methods used to assess certainty (or confidence) in the body of evidence for an outcome. | Pages 22,23 |
| **RESULTS** | | |  |
| Study selection | 16a | Describe the results of the search and selection process, from the number of records identified in the search to the number of studies included in the review, ideally using a flow diagram. | Table S4 |
|  | 16b | Cite studies that might appear to meet the inclusion criteria, but which were excluded, and explain why they were excluded. | Table S4 |
| Study characteristics | 17 | Cite each included study and present its characteristics. | Pages 22-23, Figure 1 |
| Risk of bias in studies | 18 | Present assessments of risk of bias for each included study. | Pages 22-23, Figure 1 |
| Results of individual studies | 19 | For all outcomes, present, for each study: (a) summary statistics for each group (where appropriate) and (b) an effect estimate and its precision (e.g. confidence/credible interval), ideally using structured tables or plots. | Pages 4-12 & Figures 1-5 |
| Results of syntheses | 20a | For each synthesis, briefly summarise the characteristics and risk of bias among contributing studies. | Page 6 |
|  | 20b | Present results of all statistical syntheses conducted. If meta-analysis was done, present for each the summary estimate and its precision (e.g. confidence/credible interval) and measures of statistical heterogeneity. If comparing groups, describe the direction of the effect. | Pages 4-12 |
|  | 20c | Present results of all investigations of possible causes of heterogeneity among study results. | Figures 2-5 |
|  | 20d | Present results of all sensitivity analyses conducted to assess the robustness of the synthesized results. | Page 14, Figure 7 |
| Reporting biases | 21 | Present assessments of risk of bias due to missing results (arising from reporting biases) for each synthesis assessed. | Page 15, Figure 6 |
| Certainty of evidence | 22 | Present assessments of certainty (or confidence) in the body of evidence for each outcome assessed. | Figures 1-6 |
| **DISCUSSION** | | |  |
| Discussion | 23a | Provide a general interpretation of the results in the context of other evidence. | Pages 17, 18 |
|  | 23b | Discuss any limitations of the evidence included in the review. | Page 17, 25, 26 |
|  | 23c | Discuss any limitations of the review processes used. | Pages 18, 25, 26 |
|  | 23d | Discuss implications of the results for practice, policy, and future research. | Page 19 |
| **OTHER INFORMATION** | | |  |
| Registration and protocol | 24a | Provide registration information for the review, including register name and registration number, or state that the review was not registered. | Page 27 |
|  | 24b | Indicate where the review protocol can be accessed, or state that a protocol was not prepared. | NA |
|  | 24c | Describe and explain any amendments to information provided at registration or in the protocol. | NA |
| Support | 25 | Describe sources of financial or non-financial support for the review, and the role of the funders or sponsors in the review. | Page 27 |
| Competing interests | 26 | Declare any competing interests of review authors. | Page 27 |
| Availability of data, code and other materials | 27 | Report which of the following are publicly available and where they can be found: template data collection forms; data extracted from included studies; data used for all analyses; analytic code; any other materials used in the review. | Page 27 |
